# Site-targeted mutagenesis for stabilization of recombinant monoclonal antibody expressed in tobacco (*Nicotiana tabacum*) plants

**DOI:** 10.1096/fj.15-283226

**Published:** 2015-12-28

**Authors:** Verena K. Hehle, Matthew J. Paul, Victoria A. Roberts, Craig J. van Dolleweerd, Julian K.-C. Ma

**Affiliations:** Molecular Immunology Unit, The Institute for Infection and Immunity, St. George’s, University of London, London, United Kingdom

**Keywords:** antibody engineering, degradation, proteolysis

## Abstract

This study examined the degradation pattern of a murine IgG1κ monoclonal antibody expressed in and extracted from transformed *Nicotiana tabacum*. Gel electrophoresis of leaf extracts revealed a consistent pattern of recombinant immunoglobulin bands, including intact and full-length antibody, as well as smaller antibody fragments. N-terminal sequencing revealed these smaller fragments to be proteolytic cleavage products and identified a limited number of protease-sensitive sites in the antibody light and heavy chain sequences. No strictly conserved target sequence was evident, although the peptide bonds that were susceptible to proteolysis were predominantly and consistently located within or near to the interdomain or solvent-exposed regions in the antibody structure. Amino acids surrounding identified cleavage sites were mutated in an attempt to increase resistance. Different Guy’s 13 antibody heavy and light chain mutant combinations were expressed transiently in *N. tabacum* and demonstrated intensity shifts in the fragmentation pattern, resulting in alterations to the full-length antibody-to-fragment ratio. The work strengthens the understanding of proteolytic cleavage of antibodies expressed in plants and presents a novel approach to stabilize full-length antibody by site-directed mutagenesis.—Hehle, V. K., Paul, M. J., Roberts, V. A., van Dolleweerd, C. J., Ma, J. K.-C. Site-targeted mutagenesis for stabilization of recombinant monoclonal antibody expressed in tobacco (*Nicotiana tabacum*) plants.

Plant biotechnology has become widely used for recombinant pharmaceutical protein expression (molecular pharming) since proof of concept over 25 yr ago (1). The field has advanced swiftly, with the first U.S. Food and Drug Administration drug approved in 2012: taliglucerase alfa (Elelyso), an enzyme produced in carrot cell suspension culture for treatment of Gaucher disease (2), as well as a number of products in clinical trials (3, 4). Recently, a plant-produced experimental monoclonal antibody cocktail, ZMapp, was provided for compassionate use to treat humans infected with the Ebola virus.

Monoclonal antibodies have been the focus of attention for many groups, but a number of difficulties still need to be addressed in order to maximize antibody yield from plant manufacturing systems. In particular, the quality of plant-derived IgG monoclonal antibodies can be dramatically affected by unintended proteolysis, and this has been observed in both stable transgenic plant and transient expression systems (5). Relatively little is known about the specificity of antibody degradation in plants. Western blot analysis of recombinant mAbs expressed in plants invariably revealed a number of immunoreactive bands in addition to the putative full-length antibody (5–7). Previously these bands have been explained as incomplete or partial assembly intermediates of the immunoglobulin heavy and light chains (8, 9) on the basis of previously identified assembly intermediates from murine lymphoid and malignant plasma cells (10). However, more recently, it has been demonstrated that many of the detected fragments are actually degradation products that detract significantly from the productivity of the expression system (5, 7). A variety of attempts have been made to overcome this problem—for example, by inclusion of protease inhibitors in extraction buffer or by coexpression of protease inhibitors (11–13). Protease activity *in vivo* may also be inhibited by gene silencing strategies (14). Methods for improving transcription and translation levels have been investigated (15, 16), as have methods for enhancing the stability of the product protein (17), by targeting the antibodies to specific subcellular compartments (18, 19), by glycan engineering (20) or by fusing other proteins to the antibody (21, 22).

However, none of these approaches has been able to significantly reduce proteolysis. It has been shown that recombinant antibodies, depending on their primary sequence, structural characteristics, and subcellular localization, are likely to contain amino acid sequences that are targeted by peptidases in plant cells (5, 7, 23), particularly as these heterologous proteins have never evolved in the context of the host protease environment. It has been demonstrated that there are only a limited number of plant proteolytic cleavage events in human immunoglobulin light and heavy chains, and that these were usually focused at exposed sites of interdomain regions of each immunoglobulin chain (5).

Endopeptidases show a variety of sequence specificities surrounding the cleavage site. Some cleave polypeptides at specific motifs, which in turn are characteristic of the peptidase, while others show a very broad recognition spectrum (24). For example, trypsin cleaves exclusively after Lys or Arg residues (at P1) (25). Proline usually blocks this action when found in position P1′, carboxyterminal of the scissile bond. In contrast, the plant proteases pepsin and papain have fairly broad specificity (24).

Amino acid mutations that confer resistance to proteolysis might have a measurable effect on the antibody fragmentation pattern. Expression of antibodies incorporating these mutations might therefore result in simplified antibody purification from plants and improved yields of fully assembled, functional mAbs. In the present study, an approach consisting of engineering protease resistance into antibody sequences by targeting susceptible cleavage sites was explored. Amino acids surrounding the identified cleavage sites were modified, with the aim of preventing proteolytic degradation of plant expressed mAb Guy’s 13. It was demonstrated that mutations of residues immediately proximal to identified cleavage sites modulate, but not completely eliminate, proteolytic degradation of monoclonal antibody.

## MATERIALS AND METHODS

### Transgenic plant material

Transgenic *Nicotiana tabacum* (var. Petit Havana) lines homozygous for both the γ1 heavy and κ light chain genes of the murine IgG1κ mAb Guy’s 13 (26) were used.

### Mutagenesis of mAb Guy’s 13 heavy and light chain

The γ1 heavy and κ light chain genes of mAb Guy’s 13 had previously been cloned between the *Xho*I and *Eco*RI sites of pL32, and clones, designated γ1#3 and K4.1, were used in this study (26). Using the QuikChange (Agilent Technologies, Santa Clara, CA, USA) mutagenesis protocol according to the manufacturer’s instructions, oligonucleotide primers (Supplemental Data) were used to introduce site-directed mutations. Overlapping regions of the heavy or light chain were amplified. PCR products were annealed *via* their common overlap and amplified in a second PCR reaction, then purified and ligated into plant expression vector pL32. After transformation of *Escherichia coli* XL10-Gold (Agilent Technologies), individual colonies were screened by digestion with the appropriate restriction enzymes (Supplemental Table 1) for each individual mutant. Putative mutants identified by this analytical restriction enzyme digest were confirmed by sequencing (Beckman Coulter Genomics, Bishop's Stortford, United Kingdom) before transformation of *Agrobacterium tumefaciens* EHA105.

### Transient expression in *N. tabacum* by agroinfiltration

For transient expression, the heavy and light chain genes of mAb Guy’s 13 were expressed from a plant transformation vector (pL32) (26). Wild-type (WT) *N. tabacum* plants were cultivated for 10 to 11 wk from seed. Recombinant *A. tumefaciens* cultures EHA105 harboring the light and heavy chains of Guy’s 13 were grown overnight at 28°C, with shaking at 250 rpm, in Luria Bertani medium supplemented with spectinomycin (200 μg/ml) and rifampicin (100 μg/ml). Cultures were centrifuged for 5 min at 8000 *g,* and for coinfiltration of heavy and light chains, aliquots of resuspended cell pellets (in Murashige and Skoog medium) were combined to give a total volume of 1.5 ml. The bacterial solution was injected directly using a syringe pressed firmly against the abaxial surface of a leaf (27). The plants were left to recover under standard growth conditions (temperature 25°C, 16/8 h light/dark cycle) for 5 to 7 d before leaves were harvested for analysis of the recombinant protein.

### Extraction of mAbs from transgenic and transiently expressed agroinfiltrated tobacco plants

Tissue from mature leaves of transgenic tobacco plants expressing mAb Guy’s 13 were homogenized with 3 volumes of PBS at room temperature. After 2 cycles of 20 s of homogenization using a blender (Waring Laboratory Science, Stamford, CT, USA), the plant extract was centrifuged at 17,000 *g* for 30 min at 10°C. The supernatant was passed through Whatman #3 filter paper (Whatman PLC, Maidstone, United Kingdom) and immediately placed on ice. The pH of the filtered plant juice was adjusted to 7.5 to 8.0 with 1 M NaOH and incubated for at least 30 min on ice, followed by recentrifugation at 40,000*g* for 20 min at 10°C. The supernatant was filtered through a 0.22 μm Millex GP Filter (Millipore, Consett, United Kingdom) and stored at −20°C until required. For antibody purification from agroinfiltrated *N. tabacum* plants, infiltrated leaves were sampled and homogenized for 5 min at 29 oscillations per second using a Mixer Mill MM 400 (Retsch, Haan, Germany). Samples were centrifuged at 17,000 *g* for 10 min at 10°C and the supernatant stored at −20°C until required.

For affinity purification, Protein G–Sepharose 4B resin (Sigma-Aldrich, Poole, United Kingdom) and protein A–agarose (Sigma-Aldrich) (1:1 mix) were packed into a glass chromatography column (Bio-Rad, Hemel Hempstead, United Kingdom) to give a final bed volume of ∼1 ml. Filtered supernatant was applied at a flow rate of 0.5 to 1 ml/min. The column was washed with ≥20 column volumes of PBS, and elution was with 0.1 M glycine (pH 2.5) in 1 ml fractions. Fractions were neutralized with 1 M Tris base (pH unadjusted).

To concentrate the samples, the pooled fractions were transferred to 50 ml Falcon tubes and freeze-dried under vacuum overnight. Lyophilized samples were resuspended in 200 μl dH_2_O and dialyzed overnight against PBS.

### Western blot analysis

Protein transfer was performed for 90 min onto a Hybond nitrocellulose membrane (GE Healthcare, Little Chalfont, United Kingdom) at 0.4 mA/cm^2^ and 50 V using a semidry blotting device (Bio-Rad). The membrane was incubated with 5% (w/v) nonfat milk powder (Marvel Original dried skim milk) in Tris-buffered saline (TBS) for 30 min to block nonspecific binding sites. Detection of proteins was with goat antimurine IgG, Fcγ subclass1 antiserum (115-035-205; Jackson ImmunoResearch Laboratories, Newmarket, United Kingdom), and goat antimurine IgG, κ light chain specific antisera (115-035-174; Jackson ImmunoResearch Laboratories) for 1 h at room temperature. The membrane was washed 5 times with 0.1% Tween-20 in TBS (5 min per wash), then developed using the ECL Plus Western blot detection system (GE Healthcare, Little Chalfont, United Kingdom).

### N-terminal sequencing

Purified mAb Guy’s 13 samples were separated by SDS-PAGE on 4–15% gels (Bio-Rad), blotted on PVDF membrane, and stained with Coomassie suspension G250. The N-terminal sequencing of mAb degradation fragments was performed by M. Weldon (University of Cambridge) on a Procise Protein Sequencing System (Applied Biosystems, Foster City, CA, USA).

### Densitometry

Band density from Western blot analysis was measured by densitometry (GeneTools; Syngene, Cambridge, United Kingdom). Individual infiltrations were analyzed by Student’s *t* test (*P* < 0.05) and the raw values for the fully assembled antibody compared to the raw values of all other bands present in the samples.

### ELISA

For quantification of expressed mutant antibodies or functional antigen binding ELISA, 96-well microtiter plates (NUNC MaxiSorp; Thermo Fisher Scientific) were coated for 2 h at 37°C with capture antibody–antimurine κ light chain (115-035-174; Jackson ImmunoResearch Laboratories) at 50 μl/well or with recombinant *E. coli*–derived version of SAI/II in PBS buffer normally at 5 μg/ml, respectively. The plates were washed once with distilled H_2_O before blocking with 200 μl/well PBS buffer containing 5% (w/v) nonfat milk powder for 2 h at 37°C, or overnight at 4°C. Samples were loaded at 50 μl/well, titrated accordingly, and incubated for 2 h at 37°C. Plates were then washed 3 times with distilled H_2_O containing 0.1% Tween-20 (H_2_O/T20). For detection, secondary antibody (antimurine γ chain antiserum or antimurine κ chain antiserum; Sigma-Aldrich) labeled with horseradish peroxidase (HRP) was added for 2 h at 37°C. Finally, the plates were washed 3 times with H_2_O/T20 and developed with TMB (3,3′,5,5′-tetramethylbenzidine) solution, 50 μl/well. The reaction was stopped by adding 50 μl of 2 M H_2_SO_4_ and the plates were read using a Sunrise plate reader (Tecan, Weymouth, United Kingdom) at 450 nm. To determine antibody concentrations, a standard amount of quantified, commercially available antibody was also used, and titrations were fitted with a sigmoidal dose–response curve to obtain the EC_50_ value (GraphPad Prism; GraphPad Software, La Jolla, CA, USA).

## RESULTS

### Guy’s 13 fragmentation pattern

**Figure 1.**
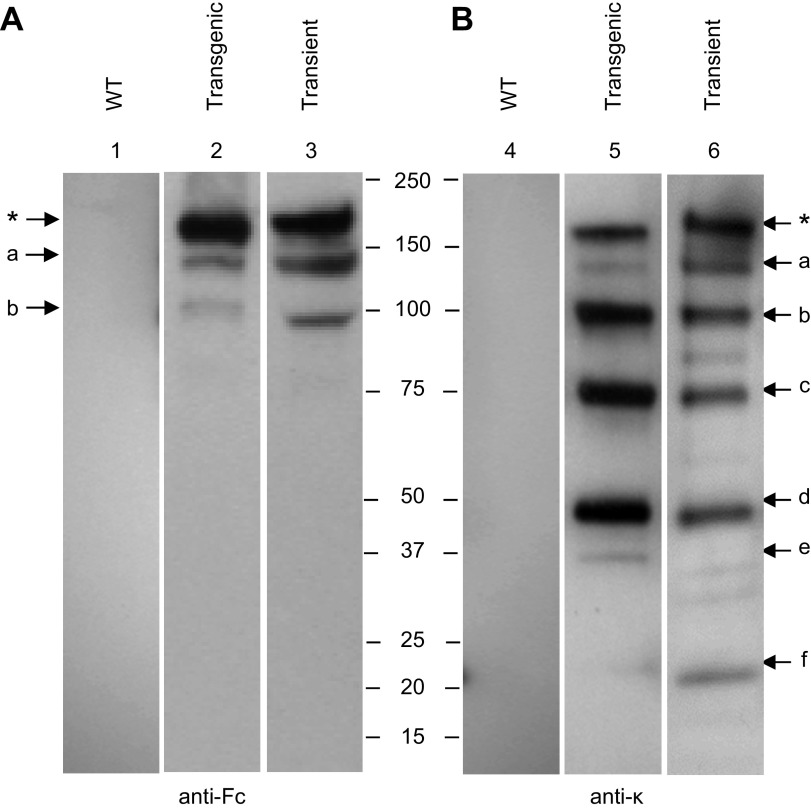
Western blot analysis of mAb Guy’s 13 expressed in *N. tabacum*. Samples from mAb expressed transgenically or transiently in *N. tabacum* were separated on nonreducing 4–15% SDS-PAGE gels, and proteins were blotted onto nitrocellulose membrane. For detection, anti-Fc (*A,* lanes 1–3) and anti-κ (*B,* lanes 4–6) antisera were used. Lanes 1 and 4, nontransgenic *N. tabacum* (WT); lanes 2 and 5, crude leaf extract of transgenic *N. tabacum* plants expressing Guy’s 13 (transgenic); lanes 3 and 6, crude leaf extract from transiently expressed mAb Guy’s 13 (transient). Asterisk represents fully assembled antibody. Letters a–f represent antibody fragments.

The murine monoclonal antibody (mAb) IgG1 Guy’s 13 was expressed by stable nuclear transformation or by transient expression in *N. tabacum*. Leaf extracts were separated by SDS-PAGE, and the presence of intact antibody as well as antibody fragments was detected by Western blot analysis (**Fig. 1**). The results indicate close similarity in the antibody fragments regardless of which expression system was used. Detection with anti-Fc antiserum (Fig. 1*A*) identified 3 major bands at *M*_r_ ∼180k (band ∗, presumed to represent fully assembled antibody), *M*_r_ ∼150k (band a), and *M*_r_ ∼100k (band b) in both of the antibody-expressing plant samples, but not the WT nontransgenic *N. tabacum* leaf extract (lane 1). A similar result was obtained using an anti–κ chain antiserum (Fig. 1*B*), although in this case more immunoreactive bands were observed. There were subtle differences between the relative intensities of individual bands—for example, bands ∗ and a are more intense in the transiently expressed sample (Fig. 1*B*, lane 6), but bands b, c, and d are more intense in the transgenic expression (Fig. 1*B*, lane 5). Some minor bands were observed in one expression system but not the other (*e.g.,* Fig. 1*B*, bands e and f). Again, the WT control *N. tabacum* demonstrated no immunoreactivity.

### Identification of N-terminal cleavage sites in mAb Guy’s 13

**Figure 2.**
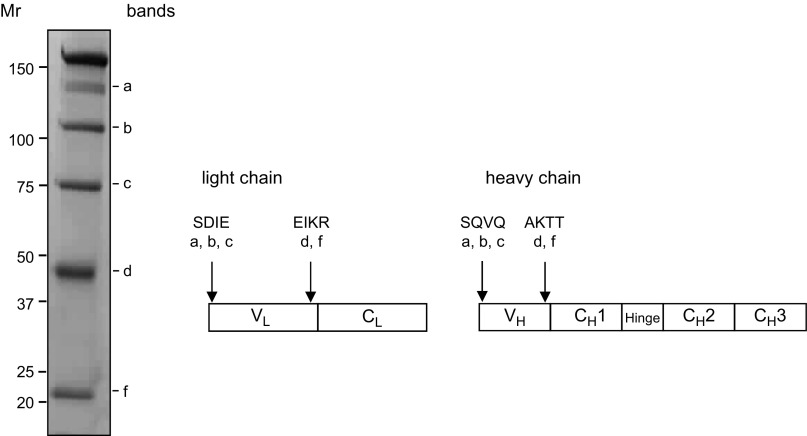
N-terminal sequencing results of nonreduced mAb Guy’s 13 fragments. N-terminal sequencing of protein A/G–purified mAb Guy’s 13 blotted onto PVDF membrane and stained with Coomassie G250. Arrows indicate position of N-terminal sequence within heavy and light chain amino acid sequence. Uppercase letters indicate sequence returned from N-terminal sequencing by Edman degradation; lowercase letters indicate major antibody fragments.

Affinity-purified Guy’s 13 fragments from a batch of transiently expressed mAb were separated by nonreducing SDS-PAGE, transferred onto PVDF membrane, and stained with Coomassie G250 (**Fig. 2**). The most prominent bands, labeled a–f, were present as before, and Edman degradation was applied to these bands to analyze the amino acid sequences of their N termini. For bands a–c, N-terminal sequences were returned corresponding to the correctly processed N termini of both the light and heavy chains. For both fragments d and f, N-terminal sequences were identified from within both the light and heavy chains. A single light chain N-terminal amino acid sequence was identified (EIKR), which resides within the junction between V_L_ and C_L_ domains. Similarly, only a single heavy chain amino acid sequence was identified (AKTT), which corresponds to the junction between V_H_ and C_H_1 domains.

Additionally, an N-terminal sequence was identified for a band detected following SDS-PAGE under reducing conditions (data not shown) at *M*_r_ ∼17k, starting with the amino acids RFSG which is found within the V_L_ region of Guy’s 13, near the boundary with C_L_.

### Mutagenesis of heavy or light chains of mAb Guy’s 13

**Figure 3.**
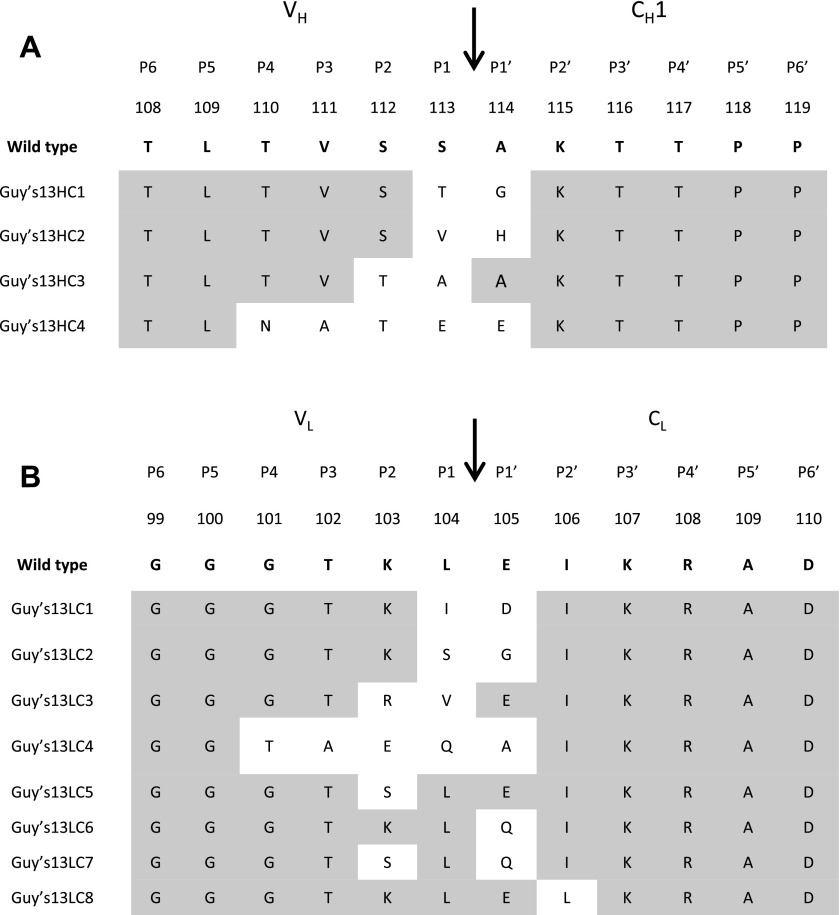
Mutants of Guy’s 13 heavy and light chain with substitutions proximal to cleavage sites AKTT and EIKR. WT Guy’s 13 heavy and light chain sequence is shown in bold with corresponding Kabat amino acid numbering. Location of protease cleavage site is indicated by arrow, and positions amino terminal (P1 to P6) and carboxyl terminal (P1′ to P6′) to scissile bond are shown at top. Amino acid sequences used in mutant heavy and light chains are shown below, with amino acid substitutions shown (unshaded). Gray shading represents unchanged amino acid residue.

Having identified a very limited number of protease-susceptible sites in the heavy and light chains of mAb Guy’s 13, a series of mutations was designed up- and downstream of the P1 and P1′ amino acids (**Fig. 3**) and expressed. Several mutagenesis approaches were considered: conservative substitution using amino acids with the same physicochemical properties; nonconservative substitution using amino acids with divergent physicochemical properties; and structurally conservative amino acid changes aimed at preserving the 3-dimensional structure of the antibody. In the first 2 cases, amino acid substitutions were made using the Kabat database (28) to assess the range of natural variants at these positions. If no possible natural variant was available in the Kabat database, changes were made using amino acids that were physicochemically most similar/or divergent to the original Guy’s 13 residue. In the last approach, *in silico* modeling, performed with the molecular graphics programs Insight (Accelrys, San Diego, CA, USA) and RasMol (29), was used to choose substitute amino acid residues, taking into account side chain interactions so as not to interfere with the tertiary structure of the antibody.

Four mutants were produced for the identified heavy chain sequence AKTT (Fig. 3), 2 representing conservative substitutions (HC1 and HC3) and 2 representing nonconservative substitutions (HC2 and HC4). Eight light chain mutants were also produced, 2 representing conservative substitutions (LC1 and LC3), 2 representing nonconservative substitutions (LC2 and LC4), and 4 representing structurally conservative substitutions (LC5 to LC8).

### Expression and characterization of IgG mAb with mutated heavy and light chains

**Figure 4.**
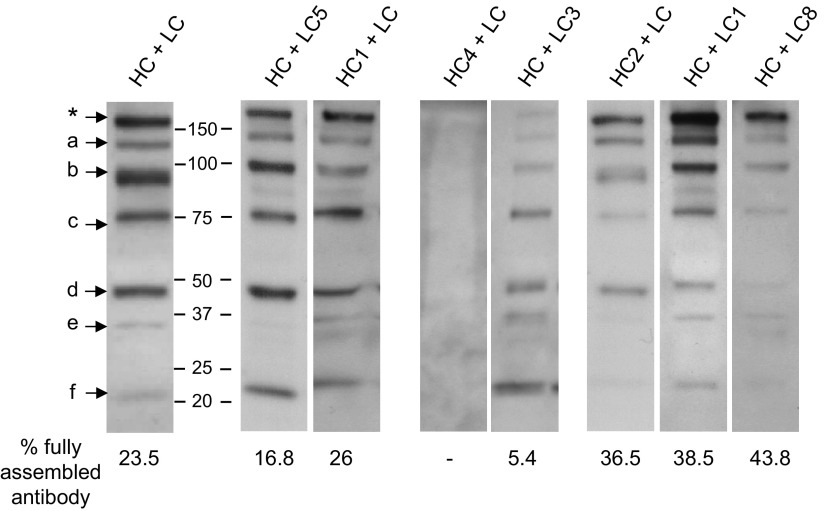
Transient expression of Guy’s 13 heavy and light chain mutants in *N. tabacum*. Nonreducing Western blot analysis of leaf extracts from transiently expressed Guy’s 13 heavy chain mutants (HC1, HC2, and HC4) expressed together with nonmutated Guy’s 13 light chain (LC) and light chain mutants (LC1, LC3, LC5, and LC8) together with nonmutated Guy’s 13 heavy chain (HC). Detection was with HRP-conjugated antimurine κ chain antiserum. Control was nonmutated Guy’s 13 heavy and light chains (HC + LC). Asterisk indicates fully assembled IgG. Letters a–f indicate major antibody fragments.

Various combinations of heavy and light chains were transiently expressed in *N. tabacum.* Extracts from infiltrated leaves were collected and analyzed by nonreducing SDS-PAGE followed by Western blot analysis with antimurine κ chain antiserum. **Figure 4** illustrates the results from 1 experiment that was representative of at least 3 repeat experiments for each mutant antibody construct. In some cases—for example, mutants LC5 and HC1—no significant differences were observed in mAb expression when the mutated chains were coexpressed with the original nonmutated corresponding immunoglobulin chain. This was confirmed by densitometry, which estimated the percentage contribution of the full-length antibody band (indicated in Fig. 4 by an asterisk) to be 16.8 and 26% respectively, compared to 23.5% for coexpressed nonmutated heavy and light chains. Similar results were obtained with mutants HC3 and LC6 (not shown)

For both HC4 (coexpressed with nonmutated light chain) and LC3 (coexpressed with nonmutated heavy chain), there was a marked reduction in full-length mAb expression. For HC4, no assembled mAb was detected, whereas for LC3, full-length mAb represented only 5.4% of the total immunodetected bands. A similar reduction in full-length mAb yield was also observed for mutants LC2 and LC4 (data not shown).

In 3 cases, an apparent increase in full-length mAb accumulations was observed even though there did not appear to be a significant overall increase in light and heavy chain expression. These were HC2 (coexpressed with nonmutated light chain), LC1 and LC8 (coexpressed with nonmutated heavy chain), and to some extent LC7 (data not shown). In the case of HC + LC1, all the bands identified in the nonmutated antibody were present, but the proportion of full-length antibody was enhanced. For HC2 + LC and HC + LC8 combinations, bands f and c, and bands d, e, and f were significantly diminished, respectively. For HC2, LC1, and LC8, the full-length mAb was the most prominent band on the Western blot analysis, representing between 36.5 and 43.8% of the total immunodetected bands. No relationship was discerned between the mutagenesis substitution strategy used and the effects on antibody stability.

The assembled HC2 mutant IgG had an increased mobility in antibody bands on membranes assessed by Western blot analysis that was not seen with other mutants (Fig. 4). Interestingly, this mobility shift affected all the major bands detected in the Western blot analysis. Under reducing conditions, no difference was observed between the relative molecular masses of HC2, nonmutated heavy chain, or other mutants (data not shown). Matrix-assisted laser desorption/ionization time-of-flight (MALDI-TOF) mass spectrometry spectra of HC2 and HC were also comparable (data not shown), indicating that the shift in migration observed for HC2 mutant antibodies was not due to a truncated heavy chain.

### Coexpression of mutated heavy and light chains of mAb Guy’s 13

**Figure 5.**
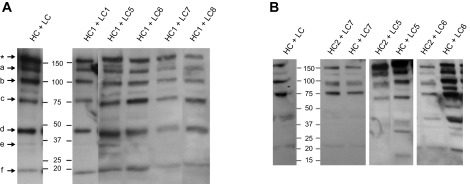
Transient coexpression of Guy’s 13 heavy and light chain mutants in *N. tabacum*. Nonreducing Western blot analysis of leaf extracts from transiently expressed Guy’s 13 heavy chain mutants (*A*) HC1 with Guy’s 13 light chain mutants (LC1, LC5, LC6, LC7, and LC8); or (*B*) HC2 with Guy’s 13 light chain mutants (LC5, LC6, and LC7). Detection was with HRP-conjugated antimurine κ chain antiserum. Control was nonmutated Guy’s 13 heavy and light chains (HC + LC). Asterisk indicates fully assembled IgG. Letters a–f indicate major antibody fragments.

IgG yield was also assessed for combinations of HC mutants with LC mutants (**Fig. 5**). Light chain mutants that had had a positive effect (LC1, LC7, and LC8) or no effect (LC5 and LC6) were coexpressed with HC1 (no effect) or HC2 (positive effect). None of the light chain mutants when coexpressed with HC1 resulted in a greater yield of intact IgG (Fig. 5*A*). This was irrespective of whether the light chain mutant itself had individually resulted in an improvement in IgG yield (Fig. 4). When HC2 mutant heavy chain was coexpressed with LC5 or LC6, there was no enhanced IgG yield (Fig. 5*B*) even though HC2 with LC (nonmutated) had previously resulted in improvement (Fig. 4). The combination of HC2 with LC7 (both associated individually with improved IgG yield) did not result in significant improvement.

**Figure 6.**
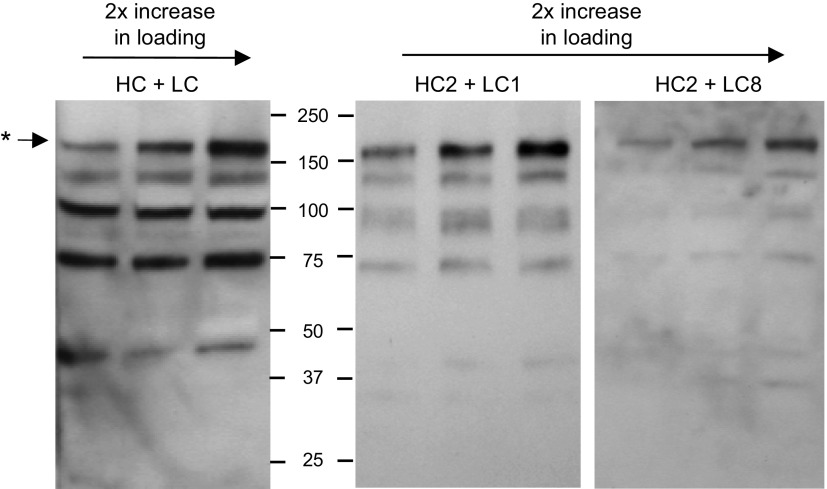
Transient coexpression of Guy’s 13 heavy and light chain mutants in *N. tabacum*. Nonreducing Western blot analysis of leaf extracts from infiltrated Guy’s 13 heavy chain mutant (HC2) coexpressed with Guy’s 13 light chain mutants (LC1 or LC8). Detection was with HRP-conjugated antimurine κ chain antiserum. Control was nonmutated Guy’s 13 heavy and light chains (HC + LC). Asterisk indicates fully assembled IgG.

However, 2 combinations of HC and LC mutants did result in significant improvement of intact antibody yield. These were HC2 with LC1 and HC2 with LC8 (**Fig. 6**). In both cases, both the heavy chain mutant (HC2) and the light chain mutants were associated individually with higher yield of intact IgG (Fig. 4). For the HC2 + LC1 combination, both bands d and f were eliminated.

**Figure 7.**
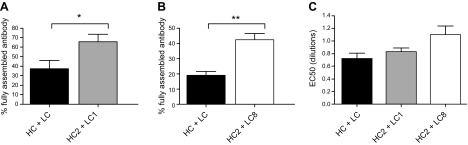
Densitometric analysis of antibody assembly from Western blot analysis. Percentage of fully assembled antibody was measured by densitometry of 9 biologic repeats for HC2 + LC1 and HC2 + LC8. Data presented as means ± sd. *A*, *B*) Independent infiltrations from *Nicotiana tabacum* of nonmutated Guy’s 13 control (HC + LC) was compared by Student’s *t* test (*P* < 0.05) to infiltrated mutants HC2 + LC1 (*A*) and HC2 + LC8 (*B*). *P < 0.05; **P < 0.005. *C*) Antigen binding activity for Guy’s 13 mutants. Equivalent amounts of each antibody preparation were serially diluted on ELISA plate coated with SAI/II. Bound antibody was detected with HRP-conjugated antimurine κ chain reagent. Absorbance values were plotted against dilutions. Titrations were fitted using sigmoidal dose–response curve (GraphPad Prism) and EC_50_ values calculated.

Replicate experiments were performed with 9 (HC2 + LC1) and 6 (HC2 + LC8) independent infiltrations, and the percentage of intact IgG was assessed by densitometry (**Fig. 7*A, B***). In each case, the nonmutated HC + LC control was infiltrated into the same plant. The data resulting from densitometry analysis confirmed the relative abundance of the fully assembled antibody to its associated fragments. All infiltrated mutant combinations (HC2 + LC1 and HC2 + LC8) showed significant differences in the percentage of fully assembled IgG compared to the IgG in the control infiltration (HC + LC). In all cases, the percentage of fully assembled antibody was approximately doubled by introducing the mutations.

To confirm that Guy’s 13 light and heavy chain mutants retained binding activity to the relevant antigen (streptococcal antigen I/II), a functional ELISA was performed (Fig. 7*C*). Here, the positive control was an extract from *N. tabacum* infiltrated with Guy’s 13 HC + LC. All antibody extracts were applied in triplicate at the same concentration, as determined previously by quantitation ELISA (data not shown). Detection of antibody bound to SAI/II was with an HRP-conjugated antimurine κ chain antibody. All mutants demonstrated a clear positive signal indicating that they were assembled and bound to SAI/II. EC_50_ dilutions were calculated for each antibody and are shown in Fig. 7*C*. There were no significant differences between the control HC + LC and any of the mutants (Student’s *t* test, *P* < 0.05).

## DISCUSSION

The development and use of recombinant monoclonal antibodies (mAbs) represents one of the leading research areas for therapeutics diagnostics and vaccine development. Alternative expression platforms such as plants are being explored to address issues of cost and scalability, especially for applications targeted to resource-poor regions. However, fully assembled antibody in plants is usually accompanied by additional immunoglobulin species of lower molecular weight, which are predominately generated by enzymatic action of plant proteases (6, 7, 23). This consistent problem reduces yields, complicates downstream processing, and increases production costs (30).

Plant proteases are often pivotal to maintaining metabolic functions, so in this study, we developed an approach of modification of specific proteolytic target sequences in a monoclonal antibody to improve expression in plants. This could be achieved without affecting antibody assembly and function. Our previous findings with other monoclonal antibodies had indicated the presence in antibodies of a limited number of conserved cleavage sites that are located in interdomain and solvent-exposed regions of the light and heavy chains (5). These findings were confirmed here using a murine IgG mAb by N-terminal sequencing of antibody fragments present in crude plant extract. In particular, an important cleavage site (EIKR) was identified in the light chain at the junction of V_L_ and C_L_, and similarly, a major site (AKTT) was found in the heavy chain at the junction of V_H_ and CH1. Whether EIKR and AKTT actually represent the exact N terminus of a newly cleaved polypeptide remains to be determined, as it is possible that primary cleavage upstream of these sites might be followed by further trimming by other proteases. Other proteolytic sites toward the C terminus of light and heavy chains are likely, and indeed predicted, from our results, but despite extensive analysis, no other new cleavage sites have been identified in the variable domains or in the constant or CH1 domains of light and heavy chains, respectively.

Previous studies have demonstrated the principle of antibody engineering to enhance stability. For example, the stability of human IgA1 against bacterial proteases was enhanced through engineering of a hybrid hinge region comprising half of human IgA1 and human IgA2 hinge region (31). Peters *et al.* (32) demonstrated that mutations introduced into the C_H_1 domain of IgG4 increased antibody thermal stability by mimicking the interchain disulfide bond arrangements observed in IgG1. In our study, a range of mutations targeted at the EIKR and AKTT sites in the antibody light and heavy chains were examined. Sequences were identified that abolished or significantly diminished full-length antibody yield, had no apparent effect, or enhanced full-length antibody yield. Although different approaches were used to design the mutations, no consistent relationship between mutation strategy and antibody yield was evident, so at this stage, a mechanism of action can only be speculated on. One possibility is that the change to a more hydrophobic amino acid in position 113 (Ser^113^Val) and a basic amino acid in position 114 (Ala^114^His) of Guy’s 13 heavy chain (mutant HC2) may alter the accessibility of the site, as both residues were found in a solvent-exposed interdomain region. Proteins typically fold in such a way as to minimize solvent exposure of the hydrophobic amino acids while exposing the hydrophilic residues. Appropriate presentation of a potential cleavage site in an exposed and unstructured region (such as a solvent-exposed loop) would be a minimal requirement for hydrolysis. The replacement of polar amino acids for hydrophobic amino acids (as done in mutant HC2) might prevent or slow down access to the cleavage site.

Of interest was the finding that an alteration in the light chain, for example, LC8, could result in the loss of multiple bands, including, apparently, those linked to heavy chain degradation. This led us to consider the likelihood that antibodies expressed in plants are cleaved by different proteases in a sequential manner. Thus, proteolysis at an initial site on the light chain might allow subsequent protease access to additional amino acid sequences previously buried within the antibody structure, including the heavy chain. Similar findings have been reported—for example, in the case of sphingosin kinase 1 (SK1) cleavage by cathepsin B. Site-directed mutagenesis of an initial target site not only abrogated a *M*_r_ 30k degradation band and stabilized intact SK1 but also reduced the appearance of an additional *M*_r_ 18k fragment (33).

An unexpected finding was that the expression of HC2 mutant combinations was associated with a consistent shift in apparent molecular mass on PAGE relative to the control (HC + LC) and molecular weight marker. The possibility of a truncated HC2 mutant, cleaved by proteases, was excluded by MALDI-TOF analysis (data not shown). A comparison of the calculated pIs for the WT HC (pI = 6.55) and the HC2 (pI = 6.59) revealed only a minor effect on the overall charge of the HC2 mutant. Changes in hydrophobicity of the HC2 mutant may explain the phenomenon of gel shifting, as Val113 is more hydrophobic than the substituted Ser113, whereas His114 is a more basic, cationic amino acid than Ala114 (34).

The next stage of this work will be to extend the analysis of protease cleavage sites to other similar antibodies in order to identify common and consistent target peptide motifs. It should ultimately be possible to perform more extensive screening of mutation strategies and to assess combination approaches that provide maximum IgG protection. The initial findings reported here for a monoclonal antibody could, of course, be extended to other recombinant proteins expressed in plants. In addition, the targeted mutation approach could be used to complement strategies that involve protease inhibition (23) and/or plant protease knockout (7) that are being adopted elsewhere, alongside multiple other approaches to maximize recombinant protein accumulation in plant systems.

## Supplementary Material

Supplemental Data

## Abbreviations

HRP: horseradish peroxidase
MALDI-TOF: matrix-assisted laser desorption/ionization time-of-flight mass spectrometry
SK1: sphingosin kinase 1
TBS: Tris-buffered saline
TMB: 3,3′,5,5′-tetramethylbenzidine
WT: wild-type
