# Supplementary material for: Site-targeted mutagenesis for stabilization of recombinant monoclonal antibody expressed in tobacco (Nicotiana tabacum) plants
Source: FASEB J. 2015 Dec 28;30(4):1590–8. doi: 10.1096/fj.15-283226 (PMC4799508; doi:10.1096/fj.15-283226)
Supplement: Supplemental Data [file supp_30_4_1590__index.html]

Site-targeted mutagenesis for stabilization of recombinant monoclonal antibody expressed in tobacco (Nicotiana tabacum) plants — Site-targeted mutagenesis for stabilization of recombinant monoclonal antibody expressed in tobacco (Nicotiana tabacum) plants — Site-targeted mutagenesis for stabilization of recombinant monoclonal antibody expressed in tobacco (Nicotiana tabacum) plants — Supplemental Data 

# Site-targeted mutagenesis for stabilization of recombinant monoclonal antibody expressed in tobacco (*Nicotiana tabacum*) plants

## Supplemental Data

- Supplemental Data
- Supplemental Data
- Supplemental Data
- Supplemental Data
