# Supplementary material for: Site-targeted mutagenesis for stabilization of recombinant monoclonal antibody expressed in tobacco (Nicotiana tabacum) plants
Source: FASEB J. 2015 Dec 28;30(4):1590–8. doi: 10.1096/fj.15-283226 (PMC4799508; doi:10.1096/fj.15-283226)
Supplement: Supplemental Data [file supp_fj.15-283226_Supplemental_Data2.docx]

**S2:** DNA primers for mutagenesis of Guy’s 13 light chain (#1-4). Complementary forward and reverse primer pairs for generating each mutant are shown, with amino acid translations shown below the nucleotide sequences. Mutated amino acid residues are shown in bold and their positions relative to the protease cleavage site are shown by P2, P1, P1’, P2’ etc. Mutations were designed to incorporate diagnostic restriction enzyme cleavage sites (underlined, and see Table S1).

1. LC1 mutant (Leu^104^Ile/Glu^105^Asp).

**VH_Guys13LC#1.1**

5’–ggaggggggaccaagAtCgaTataaaacgggctgatgctg–3’

G G G T K **I D** I K R A D A A ►

**P1 P1’**

**VH_Guys13LC#1.2**

5’–cagcatcagcccgttttatAtcGaTcttggtcccccctcc–3’

◄ A A D A R K I **D I** K T G G G

**P1’P1**

2. LC2 mutant (Leu^104^Ser/Glu^105^Gly).

**VH_Guys13LC#2.1**

5’–ggaggggggaccaagTCCgGaataaaacgggctgatgctg–3’

G G G T K **S G**  I K R A D A A ►

**P1 P1’**

**VH_Guys13LC#2.2**

5’–cagcatcagcccgttttattCcGGActtggtcccccctcc–3’

◄ A A D A R K I **G S** K T G G G

**P1’ P1**

3. LC3 mutant (Lys^103^Arg/Leu^104^Val).

**VH_Guys13LC#3.1**

5’-cgttcggaggggggaccCGgGtggaaataaaacgggctg-3’

F G G G T **R V** E I K R A ►

**P2 P1**

**VH_Guys13LC#3.2**

5’-cagcccgttttatttccaCcCGggtcccccctccgaacg-3’

◄ A R K I E **V R** T G G G F

**P1 P2**

4. LC4 mutant (Gly^101^Thr/Thr^102^Ala/Lys^103^Glu/ Leu^104^Gln/Glu^105^Ala).

**VH_Guys13LC#4.1**

5’-gtacacgttcggagggACgGccGagcAggCaataaaacgggctgatg-3’

Y T F G G **T A E Q A** I K R A D ►

**P4 P3 P2 P1 P1**’

**VH_Guys13LC#4.2**

5’-catcagcccgttttattGccTgctCggCcGTccctccgaacgtgtac-3’

◄ D A R K I **A Q E A T** G G F T Y

**P1’P1 P2 P3 P4**
