# Supplementary material for: Site-targeted mutagenesis for stabilization of recombinant monoclonal antibody expressed in tobacco (Nicotiana tabacum) plants
Source: FASEB J. 2015 Dec 28;30(4):1590–8. doi: 10.1096/fj.15-283226 (PMC4799508; doi:10.1096/fj.15-283226)
Supplement: Supplemental Data [file supp_fj.15-283226_Supplemental_Data4.docx]

**S4: Restriction Enzymes:** Diagnostic restriction sites use to select positive mutants

| Guys 13 mutants | Diagnostic restriction sites | Restriction Enzymes |
| --- | --- | --- |
| HC1 | ACCGGT | *Age*I |
| HC2 | GTGCAC | *Apa*LI |
| HC3 | CGGCCG | *Eag*I |
| HC4 | CTCTTC | *Ear*I |
| LC1 | ATCGAT | *Cla*I |
| LC2 | TCCGGA | *Bsp*EI |
| LC3 | CCCGGG | *Xma*I |
| LC4 | CGGCCG | *Eag*I |
| LC5 | ACTAGT | *Spe*I |
| LC6 | CTGCAG | *Pst*I |
| LC7 | CTGCAG | *Pst*I |
| LC8 | GAGCTC | *Sac*I |
